# Partial loss of heterozygosity events at the mutated gene in tumors from *MLH1*/*MSH2 *large genomic rearrangement carriers

**DOI:** 10.1186/1471-2407-9-405

**Published:** 2009-11-20

**Authors:** Katarina Zavodna, Tomas Krivulcik, Maria Gerykova Bujalkova, Tomas Slamka, David Martinicky, Denisa Ilencikova, Zdena Bartosova

**Affiliations:** 1 Laboratory of Cancer Genetics, Cancer Research Institute of Slovak Academy of Sciences, Vlarska 7, 833 91 Bratislava, Slovak Republic; 2 National Cancer Institute, Department of Oncologic Genetics, Klenova 1, 833 01 Bratislava, Slovak Republic

## Abstract

**Background:**

Depending on the population studied, large genomic rearrangements (LGRs) of the mismatch repair (*MMR*) genes constitute various proportions of the germline mutations that predispose to hereditary non-polyposis colorectal cancer (HNPCC). It has been reported that loss of heterozygosity (LOH) at the LGR region occurs through a gene conversion mechanism in tumors from *MLH1*/*MSH2 *deletion carriers; however, the converted tracts were delineated only by extragenic microsatellite markers. We sought to determine the frequency of LGRs in Slovak HNPCC patients and to study LOH in tumors from LGR carriers at the LGR region, as well as at other heterozygous markers within the gene to more precisely define conversion tracts.

**Methods:**

The main *MMR *genes responsible for HNPCC, *MLH1*, *MSH2*, *MSH6*, and *PMS2*, were analyzed by MLPA (multiplex ligation-dependent probe amplification) in a total of 37 unrelated HNPCC-suspected patients whose *MLH1/MSH2 *genes gave negative results in previous sequencing experiments. An LOH study was performed on six tumors from LGR carriers by combining MLPA to assess LOH at LGR regions and sequencing to examine LOH at 28 SNP markers from the *MLH1 *and *MSH2 *genes.

**Results:**

We found six rearrangements in the *MSH2 *gene (five deletions and dup5-6), and one aberration in the *MLH1 *gene (del5-6). The *MSH2 *deletions were of three types (del1, del1-3, del1-7). We detected LOH at the LGR region in the single *MLH1 *case, which was determined in a previous study to be LOH-negative in the intragenic D3S1611 marker. Three tumors displayed LOH of at least one SNP marker, including two cases that were LOH-negative at the LGR region.

**Conclusion:**

LGRs accounted for 25% of germline *MMR *mutations identified in 28 Slovakian HNPCC families. A high frequency of LGRs among the *MSH2 *mutations provides a rationale for a MLPA screening of the Slovakian HNPCC families prior scanning by DNA sequencing. LOH at part of the informative loci confined to the *MLH1 *or *MSH2 *gene (heterozygous LGR region, SNP, or microsatellite) is a novel finding and can be regarded as a partial LOH. The conversion begins within the gene, and the details of conversion tracts are discussed for each case.

## Background

Hereditary non-polyposis colorectal cancer, HNPCC (also known as Lynch syndrome), is a frequent, autosomal, dominantly-inherited predisposition mainly for early-onset of colorectal and endometrial cancers, and less frequently for other type of malignancies [1]. The clinical definition of HNPCC diagnosis is based largely on the Amsterdam criteria (AC), which were set up in 1991 with the purpose of selecting families for mutational screening of mismatch repair (*MMR*) genes, whose germline pathogenic mutations cause this condition [2,3]. The less stringent Bethesda guidelines (BG), and later, the Revised Bethesda Guidelines (RBG) were established to identify additional HNPCC patients that do not fulfill the strict Amsterdam criteria [4]. The examination of tumor tissue of HNPCC-suspected patients for the presence of microsatellite instability (MSI) and/or alterations in the MMR protein expression patterns may be also useful in pre-selecting patients for mutational screening. Nevertheless, a precise distinction between familial and sporadic MMR deficient tumors before mutational screening still remains a complex issue [5] and not all of the clinico-genetical or tumor characteristics may be available for each case.

Identification of the predisposing germline mutation is important because it confirms the clinical diagnosis of HNPCC and enables targeted clinical surveillance, which significantly reduces cancer morbidity and mortality in Lynch syndrome families [6]. Taking into account the number of potentially mutated *MMR *genes (*MLH1*, *MSH2*, *MSH6, PMS2, PMS1, MLH3*) and different types of mutations, the screening approaches are very complex and involve many techniques. Despite the tremendous advances in molecular diagnosis of HNPCC that have been made since the discovery of genetic clues of this disease, the genetic etiology of many clinically defined HNPCC patients remains unsolved because of technical limitations and/or difficulties in confirming the pathogenicity of identified gene alterations [7].

Inherited large genomic rearrangements (LGRs) in the *MMR *genes, which are not detectable by commonly applied DNA sequencing, became a subject of numerous HNPCC studies in recent years. One of the goals of these studies was to determine the frequency of these mutations in HNPCC patients. Although direct comparisons of frequencies determined in the different studies are not possible due to variability in the way the cohorts were selected, it seems that in some populations, LGRs are more frequent than in others. LGRs are reported to comprise 10% to 55% of all *MMR *gene mutations, and most occur in major *MMR *genes; i.e. the *MLH1 *and *MSH2 *genes [8-17]. An exceptionally low frequency of LGRs in these two genes (<1.5%) was reported in a study of the Spanish population [18], though a higher frequency of LGRs was found in a Basque Country population [19]. The frequency of LGRs may be remarkably higher in certain populations due to founder effects [9,10,19-24]. There is no consensus on how to select patients for LGR-screening. In many studies, the patients who were previously screened by conventional screening/scanning methods (DGGE, HDA, DHPLC, DNA sequencing), and in whom no germline mutation could be found, are analyzed for the presence of LGR [25]. However, the examination of LGR prior to the laborious exon-by exon mutation scanning of the *MMR *genes has also been suggested and is feasible by applying simple and robust techniques, such as MLPA (multiplex ligation-probe dependent amplification) [18]. Knowing the frequency of LGR in the population can significantly influence the screening algorithms for patients at risk for HNPCC.

Contrary to the germline LGRs, somatic LGRs in the *MMR *genes, which represent potential MMR inactivating events referred to as second hits during tumorigenesis, are rarely studied. The heterozygous LGR region present in the germline DNA can be used as a marker to study loss of heterozygosity (LOH) in the corresponding tumor DNA. One study showed that tumors of the *MLH1/MSH2 *germline LGR carriers often display a somatic mutation identical to one that is present in the germline DNA [1]. Furthermore, LOH analyses of these tumors using microsatellite markers flanking the respective gene have revealed that loss of the wild-type allele predominantly occurs through gene conversion, rather than mitotic recombination or physical deletion of the respective gene locus. Although in general, little is known about gene-conversion events that occur in cancer [26], it is likely that the conversion tracts do not encompass a whole sequence of the *MLH1 *or *MSH2 *gene in the LOH-positive cases. These two genes carry a number of single nucleotide polymorphisms (SNPs) that can be potentially utilized to delineate the conversion tracts more precisely than is possible using the extragenic microsatellite markers. The SNP markers, though less heterozygous than the microsatellite markers, have been proven to be useful in detecting LOH at *MMR *loci [27,28]. In their recent LOH study on the heterogeneously defined microsatellite unstable carcinomas, van Puijenbroek et al [29] used SNP arrays to assess LOH on a genome-wide level. Although, such commercial arrays are very attractive for LOH studies, they probably would not involve sufficient numbers of SNPs located within the *MMR *genes to enable a very detailed LOH study in the *MMR *gene of the LGR carriers.

To date, among 58 clinically well-defined Slovak HNPCC families, 21 clearly pathogenic *MMR *gene mutations have been identified by applying direct DNA genomic sequencing [30,31]. As DNA sequencing fails to detect LGRs, it is possible that in some of these patients, an LGR is responsible for HNPCC. In the presented study, we used the MLPA technique to screen for LGRs in the *MMR *genes for the first time in the Slovak population. As a second step, we studied potential LOH events in those patients in whom the LGR was detected in the germline, by combining MLPA and DNA sequencing at the intragenic *MLH1*/*MSH2*-SNP markers.

## Methods

### Patients and samples

This study included a total of 37 Slovak CRC patients investigated for the presence of LGR in the *MMR *genes. Eleven of these patients were enrolled in our previous study [32] while remaining 26 cases from cohort represented new patients referred to National Cancer Institute for genetic counselling after 2005. In summary, the patients were corresponding to: (a) 17 index cases of unrelated families that satisfied Amsterdam criteria I or II for HNPCC; (b) 20 unrelated index cases that satisfied Bethesda guidelines or Revised Bethesda Guidelines. The tumor specimens of 20 patients were tested for MSI (10 previously and other 10 in this study) and 18 could be analyzed for MMR protein expression by IHC (6 before and 12 now). In summary, at least one of these assays has been performed for each of 23 patients. MSI analyses in the current study were performed using new MSI Analysis System, Version 1.1 (Promega Co, Madison, WI), and the expression pattern of four MMR proteins (MLH1, MSH2, MSH6, and PMS2) was analyzed by standard IHC techniques as previously [33]. Because no pathogenic germline mutations could be detected by conventional mutation detection techniques (DGGE and/or sequencing) in the cohort, the patients were investigated for the presence of LGRs in their germline by MLPA (next section). Written informed consent was obtained from all patients included in this study. The study has been approved by The Ethics Committee of National Cancer Institute in Bratislava, and has been performed in accordance with the Declaration of Helsinki.

### LGR-screening and MLPA-based LOH analyses

DNA was isolated by either Qiagen QIAamp DNA Blood Mini kit (Qiagen, Hilden, Germany) or Recover All™ Total Nucleic Acid Isolation Kit (Applied Biosystems, Foster City, CA), depending on the source of material. For detection of aberrant exon(s) copy numbers in constitutional DNA, we used the SALSA MLPA kits MSH2/MLH1 (P003, version sold until 2006) and PMS2/MSH6 (P008) (MRC Holland, Amsterdam, The Netherlands) [34]. For MLPA-based LOH analyses in tumor DNA of *MLH1/MSH2 *LGR carriers, we used the P003 kit. After PCR amplification with 6-FAM-labeled primers, the samples were analyzed with an ABI 310 sequencer using GeneMapper software v 3.7 (Applied Biosystems, Inc., Foster City, CA). Specific peaks corresponding to each exon were identified according to their migration relative to the size standards and analyzed by MLPA-Excel spreadsheets for copy number changes. Dosage quotients equal to 0.5 (SD ≤ 20%) were considered deleted and dosage quotients 1.5 (SD ≤ 20%) duplicated. MLPA assay has been used for LOH detection by utilizing a heterozygous LGR region as a marker for LOH, similarly as described before [1,34,35], however LOH was calculated by using a formula: exon dosage quotient in the tumor DNA/exon dosage quotient in the germline DNA, that was applied on the exons affected in the germline. A value ≤ 0.5 indicated LOH at the site of large deletion and a value ≥ 1.5 would indicate an LOH of the region duplicated in the germline. The identified exon(s) copy number changes were confirmed in at least two independent MLPA reactions.

### SNP-based LOH analyses using DNA sequencing

The SNPs listed in Table 1 (n = 28) were analyzed by fluorescent capillary DNA sequencing. More SNPs (n = 18) were assessed for the *MSH2 *gene than for the *MLH1 *gene (n = 10), in order to cover large intronic regions in the central part (introns 6-8), as well as distal regions of the *MSH2 *gene. The sequences of the PCR primers used for the amplification of the regions containing SNP markers are provided in Table 2. The SNPs were visualized on an ABI PRISM 310 Genetic Analyzer. DNA sequencing at heterozygous SNP markers has been utilized for the evaluation of LOH, similarly as described previously [36,37], however for LOH assessment we used similar formula generally accepted for LOH determination by the microsatellite markers:

**Table 1:** SNP-based LOH markers used in this study.

Gene	SNP^*a*^	location	dbSNP reference ID^*b*^
***MLH1***	c.1-93G>A	Promoter	rs1800734
	c.453+79A>G	Intron5	rs4234259
	c.655A>G [p.I219V]	Exon8	rs1799977
	c.790+955C>A	Intron9	rs1558528
	c.791-1406C>T	Intron9	rs4647269
	c.791-488A>G	Intron9	rs4647277
	c.1038+86T>C	Intron11	rs2286939
	c.1039-78A>G	Intron11	rs11129748
	c.1668-19A>G	Intron14	rs9876116
	c.1990-121C>T	Intron17	rs2241031
			
***MSH2***	c.1076+1681G>T	Intron6	rs10191478
	c.1077-80G>A	Intron6	rs2347794
	c.1276+1349T>A	Intron7	rs3771272
	c.1276+1394A>T	Intron7	rs3771273
	c.1277-212T>A	Intron7	rs1981928
	c.1277-118G>A	Intron7	rs1981929
	c.1386+719T>C	Intron8	rs7602094
	c.1387-914A>G	Intron8	rs6711675
	c.1511-91G>T	Intron9	rs3732182
	c.1511-9A>T	Intron9	rs12998837
	c.1661+12G>A	Intron10	rs3732183
	c.1759+107A>G	Intron11	rs3764959
	c.1759+183G>A	Intron11	rs3764960
	c.1760-1207C>T	Intron11	rs3821227
	c.2006-6T>C	Intron12	rs2303428
	c.2210+175G>A	Intron13	rs4583514
	c.2210+274T>G	Intron13	rs4608577
	c.2635-214T>C	Intron15	rs2042649

^*a *^SNP nomenclature reflecting the recommendations of Human Genome Variation Society; ^*b*^According to the dbSNP at http://www.ncbi.nlm.nih.gov/SNP/index.html. For the determination of SNP location, the cDNA sequence alignements have been performed using NCBI RefSeq NM_000249.2 with genomic contigs NT_005580.6 and NT_022517.17 for the *MLH1*, and NM_000251.2 with genomic contig NT_022184.14 for the *MSH2 *gene.

**Table 2:** Primers for amplification of the fragments containing SNP markers.

SNP	Forward primer sequence (5' → 3')	Reverse primer sequence (5' → 3')	bp^*a*^
**Gene *MLH1***			

**c.1- 93G>A**	CAACCCACAGAGTTGAGAAATTT	GCTCAACGGAAGTGCCTT	152
**c.453+79A>G**	TGGAAAACTGAAAGCCCC	TTAGAGGATATCTTGGGACCTCC	201
**c.655A>G**	CGTGGACAATATTCGCTCC	CTAAAGCAAACTCTTAACACACATAATATC	156
**c.790+955C>A**	CCAGCTGTAAATGTTTCAAAATAT	AACATTTTCTGAGCCTTGATTAAA	200
**c.791- 1406C>T**	CAGAGTACAGTGACATTAATGAT	TCTCTCTGAACAGATTTAGATGA	183
**c.791- 488A>G**	TGATACCATTTCTGTGCCATTTT	GGCCAGCACTATTATAGACGT	157
**c.1038+86T>C**	GTCAGGGCGCTTCTCATC	GGAACAACAGCACAATACCTT	173
**c.1039- 78A>G**	ATAATTTTAAGATTATAAGGATTTTG	GTGGAGAGACTCAGAATAAGA	186
**c.1668- 19A>G**	ACAGCCAGGCAGAACTATTTC	TGAGTATCTGGTAGAACAGTTCTTC	153
**c.1990- 121C>T**	CCTGCTCTCATCCCCACT	ACCTGGTCCAAAGAAATTCA	178

**Gene *MSH2***			

**c.1076+1681G>T**	AACGATATTGGAATGATTGGATC	ACTTGCAAATTACCCAGTCCTT	177
**c.1077- 80G>A**	AAATGGAATTTTGAGCTGATTT	ATCTTCTACAAAAGCTTCCACTAA	134
**c.1276+1349T>A**	GTTGTTATGTCTAATACATAAAGC	TCAAAACGTATGTTTTAATGGTTC	157
**c.1276+1394A>T**	GTTGTTATGTCTAATACATAAAGC	TCAAAACGTATGTTTTAATGGTTC	157
**c.1277- 212T>A**	CATGTTTCTGCATCTATATTACTT	TAAACAAGCATCATTTGATCCAAA	201
**c.1277- 118G>A**	CATGTTTCTGCATCTATATTACTT	TAAACAAGCATCATTTGATCCAAA	201
**c.1386+719T>C**	TCAGTCTTAGCCTCCCAAAAT	CGACAACTATTTAGGAGATGCA	131
**c.1387- 914A>G**	AGTCTCAGTATGTCACCTAGG	TAGCAGTGCGCACCTGTAGT	131
**c.1511- 91G>T**	CAAGTAAGTAGTATTTGAATCTTTTCTG	GCACTGGAATCCAGTTTAATCT	202
**c.1511- 9A>T**	CAAGTAAGTAGTATTTGAATCTTTTCTG	GCACTGGAATCCAGTTTAATCT	202
**c.1661+12G>A**	TCCAGAAGAATGGTGTTAAATTT	TTAGTAAAACTTATCATAGAACATTCAC	120
**c.1759+107A>G**	GGCTTTTGGTAACAGAAGAAAAA	CTGCCATTTTTTGTTTCTATGTG	176
**c.1759+183G>A**	GGCTTTTGGTAACAGAAGAAAAA	CTGCCATTTTTTGTTTCTATGTG	176
**c.1760- 1207C>T**	TAATTAGGTCTGCTTGGCCATT	CAGAACATTCAAGATAAAAGCATT	166
**c.2006- 6T>C**	ACATCTTTGGGCAGGCTG	CCATGAGTACTATCACCCCA	214
**c.2210+175G>A**	GTCTTAGTTTAATAGTTGTTTTCC	ATTCCAGGAAGTGTGAACAGT	198
**c.2210+274T>G**	GTCTTAGTTTAATAGTTGTTTTCC	ATTCCAGGAAGTGTGAACAGT	198
**c.2635- 214T>C**	CCAGTATTCTTTGTAAACCTTGA	GTCTCAAACTCCTCCCACATT	156

^a ^size of amplicon in base pairs (bp); Note: several SNPs are located on the same amplicon

The alleles with higher intensity of signal were set for N1 (in normal DNA) or T1 (in tumor DNA) while the alleles with lower intensity of signal were set for N2 (in normal DNA) or T2 (in tumor DNA), and ratios ≤ 0.5 implied the presence of LOH at the respective SNP marker.

### The definition of partial LOH

The concurrent retention of heterozygosity of at least one marker and loss of heterozygosity of at least one other marker within the gene.

## Results

### Screening of the LGRs in the germline DNA

**Figure 1:**
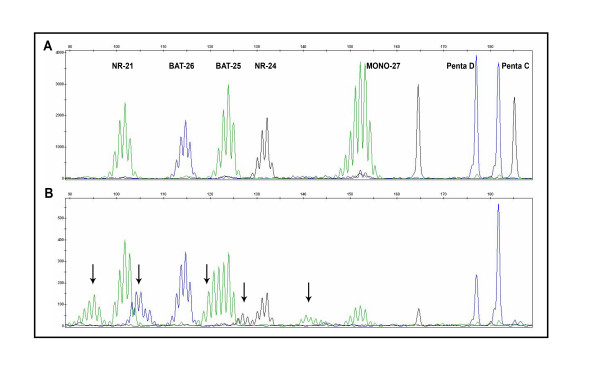
**MSI analysis**. **A**: Normal DNA. **B**: Matching tumor DNA with MSI-H status. The presence of new alleles in the tumor sample (arrows) that were not present in the normal sample indicates microsatellite instability. The distribution of MSI markers and stable pentanucleotide markers is indicated in upper part of panel A.

**Figure 2:**
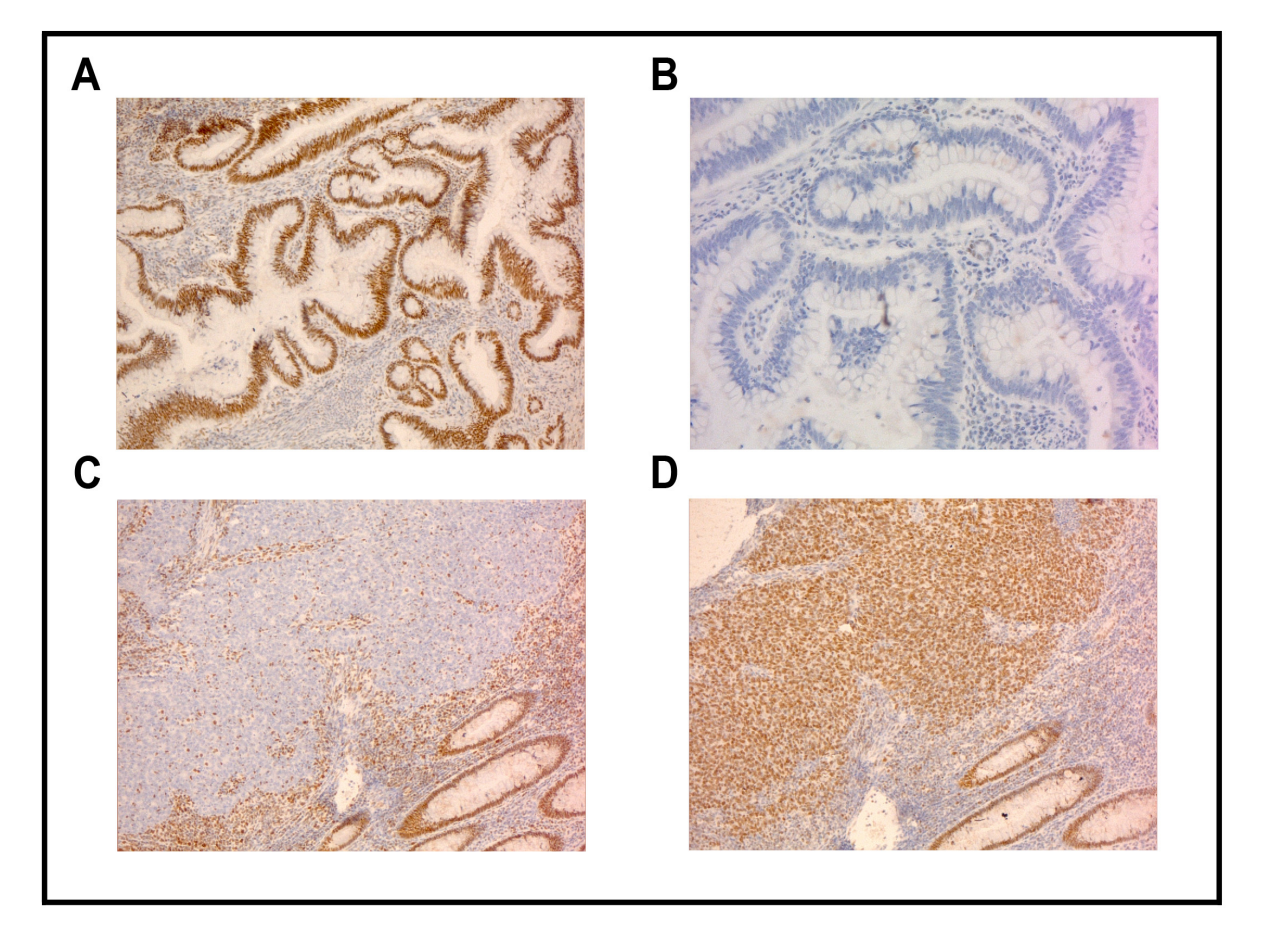
**IHC analyses**. **A**: An example of tumor with positive staining for MLH1. **B**: Same tumor as in A, with negative staining for MSH2. **C**: An example of tumor with negative staining for MLH1. **D**: Same tumor as in C, with positive staining for MLH1.

The cohort of 37 index patients at risk for HNPCC screened in this study for LGRs has been established mainly on the basis of clinico-genetical criteria for HNPCC (AC-I and II, BG and RBG) and a lack of a germline mutation in the major MMR genes (*MLH1 *and *MSH2*) by conventional mutation screening techniques. Eleven of these patients were extensively described in our previous study; the tumor specimens of 10 of them were analyzed by MSI assay in which 8 and 2 showed MSI-H and MSI-L status, respectively [32]. In the same study, 6 tumors were analyzed by IHC in which 2 analyses failed due to fixation problem and from 4 successfully stained tumors, 3 had a loss of MSH2 expression while one was MLH1 negative [32]. From 26 new cases, the tumor specimens of 10 of them were analyzed by MSI assay in which 9 and one showed MSI-H and MSI-L status, respectively. Figure 1 shows the representative pictures from these analyses. We have not included patients with stable tumors (MSS) in the study, however the MSI status for 17 cases from our cohort could not be analyzed because of no availability of fresh or FFPE tumor tissue. Figure 2 shows the representative pictures from IHC analyses. Twelve tumors were stained; 1 analysis failed due to fixation problem and from remaining 11 successfully stained tumors, 3 showed loss of MSH2 expression while eight were MLH1 negative. Because no pathogenic germline mutations could be detected by conventional mutation detection techniques in cohort, the patients were subjected to LGR screening by MLPA. In total, 7 LGRs were identified, all of which were detected using the SALSA MLPA kit P003 MSH2/MLH1. No deletions or duplications at the exons of other *MMR *genes detectable by SALSA MLPA kit P008 were observed (all exons of *MSH6 *and *PMS2*, and some exons of *MLH1*, *MLH3 *and *MSH3*). The clinico-pathological features of the LGR carriers and descriptions of identified changes are summarized in Table 3. Only one LGR was found in the *MLH1 *gene (in the patient SK-22)- a deletion of exons 5 and 6. LGRs found in the *MSH2 *gene represented a single duplication of exons 5 and 6 (SK-20 case) and three different types of deletions in five patients: an exon 1 deletion (SK-23), a deletion of exons 1-3 (SK-21 and SK-28), and a deletion of exons 1-7 (SK-14 and SK-25). Most patients with detected LGRs (5/7) fulfilled strict Amsterdam criteria for HNPCC. The two remaining LGR carriers complied with the less stringent Bethesda Guidelines. The average age of the LGR carriers at diagnosis was 38 years and three of them suffered from synchronous cancers. The MSI status of the tumors of five LGR carriers was known from our previous study [32]. These were SK-14, SK-20, SK-21, SK-22 with MSI-H tumors, and SK-23 who had low-level MSI cancer. The tumor tissue of the SK-25 case was not available for MSI analysis, however MSI-H status in the tumor of SK-28 patient was determined in recent study. The absence of MSH2 protein in tumors of the SK-20 and SK-21 cases, and a loss of the MLH1 protein expression in the SK-22 tumor detected by the IHC evaluation of tumor sections previously was in agreement with MLPA findings in this study. The mutations identified in five index patients were also present in 7 out of 10 tested relatives.

**Table 3:** Germline LGRs identified in Slovakian HNPCC families.

Family code	HNPCC criteria	Tumor localization (age at diagnosis)	Gene	Deleted or duplicated exons (systematic nomenclature)	5' deletion	MSI	IHC	LOH
SK-14	AC-I	ascendens + sigmoid colon (27)	*MSH2*	del1-7 (c.1-?_1276+?del)	15 kb	MSI-H	*	partial
SK-20	AC-II	sigmoid colon (36)	*MSH2*	dup5-6 (c.793-?_1076+?dup)	*n.a.*	MSI-H	MLH1^+^, MSH2^-^	partial
SK-21	BG	cecum (27)	*MSH2*	del1-3 (c.1-?_645+?del)	15 kb and 27 kb	MSI-H	MLH1^+^, MSH2^-^	none
SK-22	AC-I	sigmoid colon (36)	*MLH1*	del5-6 (c.381-?_545+?del)	*n.a.*	MSI-H	MLH1^-^, MSH2^+^	partial
SK-23	AC-I	cecum + ascendens colon (53)	*MSH2*	del1 (c.1-?_211+?del)	**-**	MSI-L	*	none
SK-25	AC-I	cecum (42)	*MSH2*	del1-7 (c.1-?_1276+?del)	15 kb	#	#	
SK-28	BG	ascendens colon + rectum (48)	*MSH2*	del1-3 (c.1-?_645+?del)	*n.d.*	MSI-H	*	n.i.

Fulfillment of HNPCC criteria: AC-I, AC-II, Amsterdam criteria I, II; BG, Bethesda guidelines. Systematic nomenclature: According to the recommendations of the Human Genome Variant Society using reference sequences NM_000249.2 (*MLH1*) and NM_000251.1 (*MSH2*). 5' deletion: the probes detecting regions located 15 or 27 kb before *MSH2 *localized in the *TACSTD1 *gene; *n.a*., not applicable; - no reduction of the signal at any of the *TACSTD1 *probes, *n.d*., not determined; MSI, microsatellite instability analysis: MSI-H, high level of MSI; MSI-L, low level of MSI; IHC, immunohistochemical analysis, ^+^, a positive staining, ^-^, a negative staining, * staining failed due to fixation problem; #, no FFPE tissue available for analysis; LOH, loss of heterozygosity, n.i. not informative in any LOH marker

### Further characterization of identified LGRs

In the MLPA assay, mutations and/or polymorphisms that are very close to the probe ligation site may also result in a reduced relative peak area. Therefore, deletions detected by a single probe (single exon deletions) always require confirmation. In order to confirm *MSH2 *deletion of exon 1 in the SK-23 case, we used the SALSA MLPA kit P008 PMS2/MSH6 including a single probe to *MSH2 *exon 1, different from the exon 1 probe present in the SALSA MLPA kit MSH2/MLH1 (P003). We again observed a reduction of peak area at exon 1 in the SK-23 case using the second kit, thus confirming the presence of this single exon deletion. The remaining four cases carrying multi-exonic *MSH2 *deletions involving exon 1 also displayed a reduction of peak area at the probe for *MSH2 *exon 1. In addition (as shown in Table 3), three patients exhibited an aberrant hybridization signal for one or two probes, which were confined to the *TACSTD1 *gene located upstream of *MSH2*.

### LOH analyses in tumors from LGR carriers

**Figure 3:**
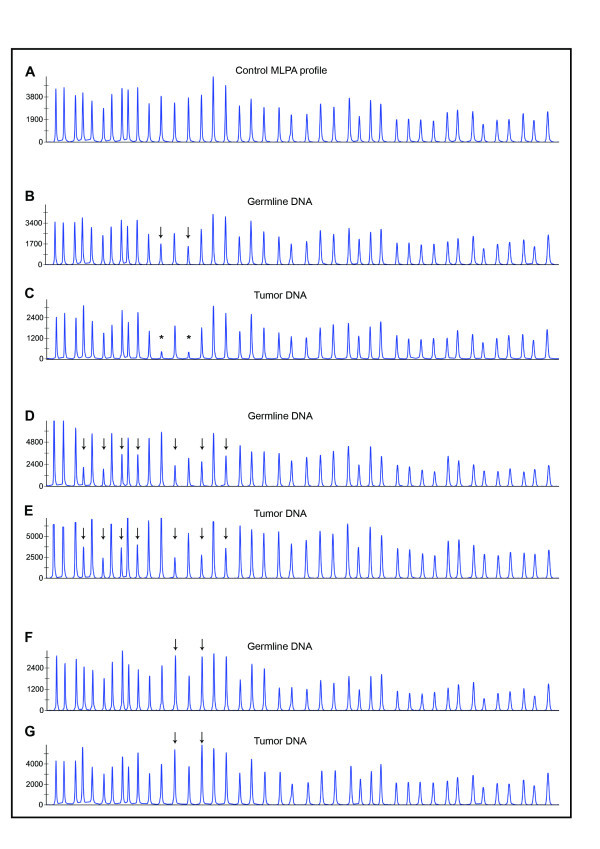
**MLPA-based LOH analyses**. **A**: The *MSH2*/*MLH1*-MLPA analysis profile of healthy control DNA. **B**: germline del5-6 *MLH1 *(SK-22 patient). **C**: SK-22 tumor, homozygous del5-6 of *MLH1 *indicating LOH at LGR. **D**: SK-14 germline, *MSH2 *del1-7. **E**: SK-14 tumor, retention of heterozygosity at LGR region. **F**: SK-20 germline, dup5-6 *MSH2*. **G**: SK-20 tumor, retention of heterozygosity at LGR region. Arrows, deleted/duplicated exons; asterisk, LOH.

**Figure 4:**
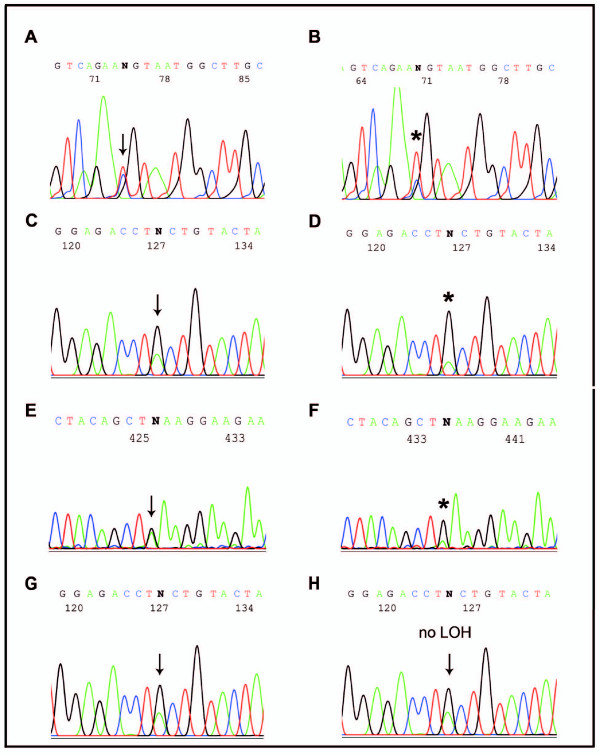
**SNP sequencing-based LOH analyses**. **A**: Germline DNA of SK-14 at the c.1759+107A>G (Intron11, *MSH2*), reverse sequencing. **B**: SK-14 tumor DNA showing LOH at the c.1759+107A>G (Intron11, *MSH2*), reverse sequencing. **C**: Germline DNA of SK-20 at the c.1277-118G>A (Intron7, *MSH2*), forward orientation. **D**: SK-20 tumor DNA showing LOH at the c.1277-118G>A (Intron7, *MSH2*). **E**: Germline DNA of SK-22 at the c.1-93G>A (Promotor, *MLH1*), forward orientation. **F**: SK-22 tumor DNA showing LOH at the c.1-93G>A (Promotor, *MLH1*). **G and H**: An example of DNA sequencing profiles of paired germline and tumor DNA presenting no LOH at the c.1277-118G>A (Intron7, *MSH2*) in the tumor. Note: LOH was calculated as described in Methods.

Six tumors from different LGR carriers were available for LOH analyses. MLPA revealed a somatic copy deletion identical to the germline deletion (*MLH1 *del5-6) in the SK-22 tumor (Figure 3B, C). Other tumors did not exhibit LOH at the heterozygous LGR region (two examples are shown on Figure 3D, E and 3F, G). Interestingly, as in the case of LOH at the LGR (SK-22), in the previous studies from our laboratory we have seen a retention of heterozygosity at intragenic microsatellite marker D3S1611 located in *MLH1 *intron 12 [32,38] [see additional file 1]. The case of retention of heterozygosity at one informative marker with a loss of heterozygosity at another informative marker within the same gene was subsequently termed 'partial LOH'. The observation of partial LOH in the *MLH1 *gene in the SK-22 tumor led us to search for SNP markers that are heterozygous/informative in our samples and enable a detailed examination of LOH within the mutated *MLH1 *or *MSH2 *gene. By testing the SNP markers listed in Table 1, we detected additional LOH events in three tumors. Germline DNA of the SK-22 case was heterozygous at the c.1-93G>A locus located in the promoter of the *MLH1 *gene and displayed LOH at this SNP (Figure 4E and 4F), indicating that the conversion covered not only the LGR region but also the region upstream of the LGR. The remaining two cases, neither of which showed LOH at the LGR region, exhibited LOH at the informative SNP markers (Figure 4A-D). The tumor of the SK-14 patient, which retained heterozygosity at *MSH2 *del1-7, displayed LOH at four SNPs (c.1511-91G>T and c.1511-9A>T in intron 9, c.1661+12G>A in intron 10, and c.1759+107A>G in intron 11), indicating the presence of a partial LOH located downstream of the LGR region. Germline DNA of the SK-20 patient was heterozygous at the c.1277-118G>A locus in intron 7 and showed LOH at this SNP. We had previously detected LOH by SNaPshot in the same case at two *MSH2*-SNPs located in intron 1 (c.211+9C>G, c.211+98T>C), which are difficult to sequence due to formation of secondary structures in the respective regions [28]. Taken together, two distinct LOH events interrupted by heterozygous dup5-6 (tandem arrangement assumed) are apparently present in the tumor of the SK-20 patient. In two cases (SK-21 and SK-23), no LOH was detected at any informative SNP, and the SK-28 germline DNA was SNP-uninformative. It has to be noted that for LOH measurements of each patient sample pair of normal and tumor tissue, the same tumor DNA isolation samples were analyzed in different assays taking in account of tumor heterogeneity, including the case SK-22 analyzed in a different time in a different study by microsatellite marker D3S1611. All data used for LOH analyses by MLPA and SNP markers together with calculated LOH ratios are summarized in additional files 2 and 3. There was no apparent difference of the clinico-pathological characteristics between the tumors with partial LOH (SK-14, SK-20, SK-22) and tumors without LOH (SK-21, SK-23).

## Discussion

The index cases of 37 families included in this study were analyzed for LGRs in the *MMR *genes using two MLPA kits, and 7 (19%) were found to have this type of alteration. Taking into account all mutations identified so far (28 mutations in total), including the mutations found in this study, the LGRs account for 25% of clearly pathogenic changes in *MMR *genes from Slovakian HNPCC families. The large deletion in the *MLH1 *gene, a del5-6, has not been reported in other populations and, as a single mutation, represents only 6.7% (1 of 15) of all *MLH1 *mutations, while LGRs in the *MSH2 *gene account for 50% (6 of 12) of all *MSH2 *mutation-positive cases [30-32] (one family with inherited *MSH6 *mutation is unpublished). Although large duplications in *MMR *genes seem to occur much less frequently than deletions [18,39-43], we uncovered one duplication of exons 5 and 6 affecting the *MSH2 *gene in our relatively small cohort. A duplication of these two exons in the *MSH2 *gene has been reported [14].

Our results provide the first evidence that, like in many other studied populations, large genomic changes in the *MLH1/MSH2 *genes exist in Slovakian HNPCC families. In addition, this type of mutation is frequent, particularly in the *MSH2 *gene, implying that the mutation screening algorithm should begin with MLPA and not with DNA sequencing, especially in cases where the protein expression pattern of the tumor shows a loss of MSH2 protein or is unknown. For cases that lack detectable MLH1 protein expression in tumors analyzed by IHC, DNA sequencing of the *MLH1 *gene prior MLPA screening is more appropriate. LGR correlated strongly with MSI-H status; more than 83% tumors of LGR carriers displayed MSI-H. However, also high rate of AC-I patients with MSI-L tumors may be potentially LGR carriers (in our cohort ~33%). For patients with no FFPE tissue available for MSI analysis such as the case SK-25 in our study, MLPA assay may be considered if the patient fulfills strict AC criteria. Similarly, MMR gene expression results correlated with MLPA findings, indicative of *MSH2 *and *MLH1 *mutation.

Similar to what has been found in other studies, there was a high proportion 5/6 (83%) of deletions encompassing exon 1 among the *MSH2 *rearrangements [8,11,13,41,42,44,45]. Common origin, or founder effect, cannot be excluded for deletions of exons 1-3 detected in the patients, SK-21 and SK-28, and likewise of exons 1-7 identified in the patients, SK-14 and SK-25. The examination of haplotypes and/or breakpoints may help to determine whether this is the case. Unfortunately, the risk haplotype could not be traced in the families due to the small number of people available for examination. For deletions encompassing exon 1, determination of breakpoints by long range PCR is difficult given that 5' breakpoints may be located up to 200 kb upstream of the *MSH2 *transcription initiation site [44]. However, according to the preliminary results of the large on-going study to test a new methodological approach for breakpoint detection involving the LGR carriers of our work, the SK-14 and SK-25 cases do seem to share common breakpoints (Dr. Benno Röthlisberger, personal communication). For three out of five *MSH2 *deletion cases (SK-14, SK-21, and SK-25), we demonstrated that one breakpoint should lie in the *TACSTD1 *gene, which is upstream of the *MSH2 *gene. Current findings that some HNPCC patients carry aberrations exclusively in the *TACSTD1 *gene leading to the generation of *TACSTD1/MSH2 *fusion transcripts [46,47] led to the creation of a new version of the P003 kit (B1 version), including *TASCTD1 *probes to enable simultaneous MLPA analysis of this gene and the major *MMR *genes (*MLH1 *and *MSH2*). Using the P008 kit (MSH6/PMS2) in our cohort, we detected copy number changes for the *TASCTD1 *gene only in conjunction with deletions of exon(s) in the *MSH2 *gene.

Although based on a small number of cases, our cumulative data from LOH analyses performed by various markers (heterozygous LGR region, SNPs, and microsatellites) indicate that partial LOH can take place at the mutated *MLH1 *or *MSH2 *loci in LGR carriers. We have adapted the term 'partial LOH' from LOH studies conducted on the chromosomal level, showing for instance partial LOH confined to one arm of a chromosome [48], and have used this term to signify concurrent retention of heterozygosity of at least one marker and loss of heterozygosity of at least one other marker within the gene. At this point, it is not clear whether partial LOH also occurs in HNPCC tumors of individuals carrying other types of *MMR *gene mutations, for instance point mutations. A varying frequency of LOH has been described in the literature for the *MLH1 *and *MSH2 *loci in the series of *MMR *gene mutation carriers. LOH at the *MLH1 *locus has been reported in 35-85% of all tumors with a germline mutation in the *MLH1 *gene [49-55]. LOH at the *MSH2 *locus has been described in 14-50% of all tumors with a germline *MSH2 *mutation [49,51,54,56]. Given that partial LOH may be not detected when only a few markers are used, it is possible that the importance of LOH events at *MMR *genes in HNPCC tumors is still underestimated.

**Figure 5:**
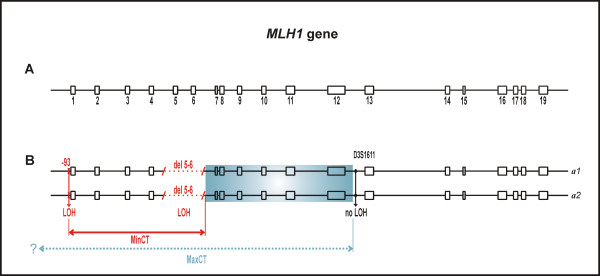
**Schematic representation of partial LOH in the *MLH1 *gene**. **A**: The structure of the *MLH1 *gene with 19 exons (open boxes numbered 1-19). Exon and intron (solid line) sizes are drawn approximately to scale. **B**: The scheme of two *MLH1 *alleles (*a1, a2*) in the tumor of patient SK-22 displaying LOH at SNP marker c.1-93G>A, homozygous del5-6 (LOH), and retention of heterozygosity at the microsatellite marker, D3S1611, located in intron 12. MinCT, minimal converted tract; MaxCT, maximal converted tract; the initiating point of putative gene conversion can lie anywhere within the blue colored box; question mark indicates that extension of MaxCT remains unknown. Note: The term 'initiating point' is not used in the sense to indicate direction of gene conversion.

**Figure 6:**
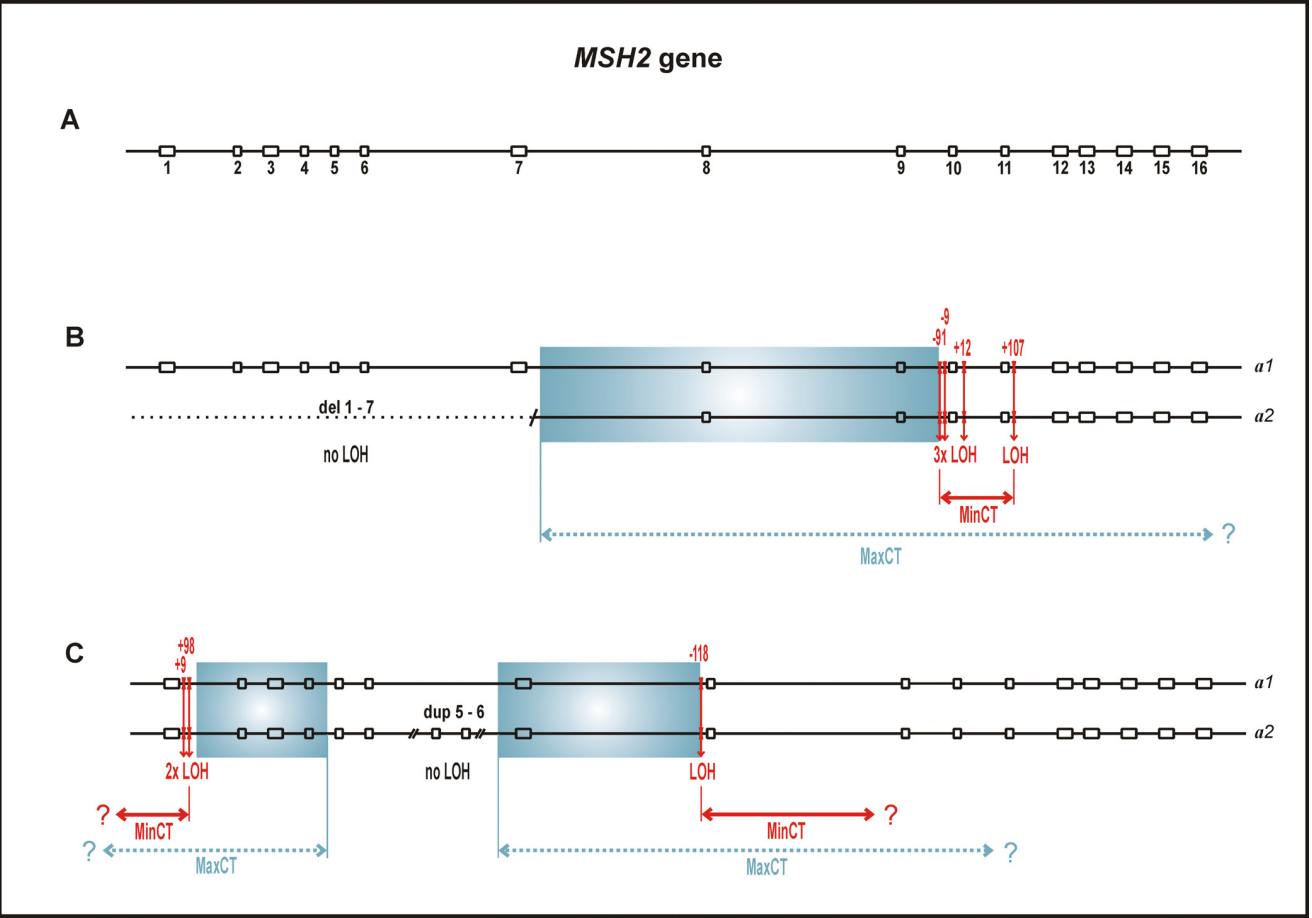
**Schematic representation of partial LOHs in the *MSH2 *gene**. **A**: The structure of the *MSH2 *gene with 16 exons (open boxes numbered 1-16). Exon and intron (solid line) sizes are drawn approximately to scale. **B**: The scheme of two *MSH2 *alleles (*a1, a2*) in the tumor of patient SK-14 displaying retention of heterozygosity (no LOH) at del1-7 (dotted line) and LOH at SNP markers c.1511-91G>T, c.1511-9A>T, c.1661+12G>A, and c.1759+107A>G. **C**: The scheme of two *MSH2 *alleles (*a1, a2*) in the tumor of patient SK-20 displaying retention of heterozygosity (no LOH) at dup5-6 (presumably tandemly arranged) and LOH at SNP markers c.211+9C>G, c.211+98C>T, both in intron 1, and c.1277-118G>A in intron 7. MinCT, minimal converted tract; MaxCT, maximal converted tract; the initiating point of putative gene conversion can lie anywhere within the blue colored box; question mark indicates that extension of MaxCT and MinCT remains unknown. Note: The term 'initiating point' is not used in the sense to indicate direction of gene conversion; LOH status in the SK-20 tumor (C panel) at the c.211+9C>G and c.211+98C>T loci has been observed previously [28].

Schematic representations of partial LOHs in both genes are shown in Figure 5 and 6. The precise characterization of gene conversion events through the determination of initiating and terminating points is practically difficult; thus, annotation of the converted tract usually represents the minimal and maximal converted tracts (MinCT, MaxCT) [26]. In the tumors we examined, the overall heterozygosity of SNP markers was unexpectedly low, and thus allowed us to characterize converted tracts only partially. The microsatellite markers flanking the *MLH1 *or *MSH2 *gene that were tested in previous studies from our laboratory [32,38] were also uninformative, due to the presence of instability in these MMR deficient tumors.

The underlying mechanism for partial LOH is unclear at the moment; however, it is likely that specific motifs surrounding DNA sequences involved in gene conversion [26] play a role in partial LOH. Such motifs include alternating purine and pyrimidine or polypurine and polypyrimidine tracts, minisatellite sequences, and chi-like sequences, all of which are present in the *MLH1 *and *MSH2 *genes. The only two chi sequences (5'-GCTGGTGG-3') present within the *MLH1 *gene, as well as the (TA)_12_(T)_21 _tract known as BAT-21, are coincidentally located in the region that we confined to the initiating point of the putative gene-conversion event in the SK-22 tumor (blue colored box in Figure 5). The regions of the *MSH2 *gene that we confined to the initiating points of the gene-conversion events in the SK-14 and SK-20 tumors are rich of polypyrimidine tracts (blue colored box in Figure 6). The *Alu *sequences, which are abundant in both *MMR *genes, are also considered to play a role in gene conversion; however, their action is attributed mainly to interlocus, rather than interallelic, gene-conversion events [26].

Understanding the nature of the second, i.e. somatic, hit in the respective *MMR *gene may be crucial for the understanding of cancer initiation in HNPCC patients. Interestingly, only in the case of SK-22 carrying an exon 5-6 del of the *MLH1 *gene, has the wild-type sequence been replaced by the mutated sequence encompassing a deletion of exons 5 and 6 and resulting in a homozygous alteration in the tumor. This LOH event should clearly lead to a complete inactivation of the *MLH1 *gene in the tumor which is in correlation with MSI and IHC findings. In the remaining two cases of *MSH2 *mutation, it is difficult to distinguish which allele served as the 'donor' (unaltered) and which as the 'acceptor' (altered). Since the replaced region does not harbor a genomic rearrangement observed in the germline, the role of such a 'second hit' in tumorigenesis remains unclear. Nevertheless, in one tumor (SK-20) that could be analyzed by MSI and IHC, presumably MMR inactivation had occurred as demonstrated by the presence of high level MSI (MSI-H) and loss of MSH2 expression. It cannot be excluded that the gene conversion-mediated sequence homogenization at several SNPs plays a role in tumorigenesis. Another explanation might be the presence of a 'third hit', which is difficult to fully assess in tumor-derived DNA. Clearly, future studies on larger sets of HNPCC patients with various types of mutations are needed to confirm the occurrence of partial LOH events in the *MMR *genes of HNPCC tumors and to elucidate the mechanism by which they arise and their roles in tumorigenesis.

## Conclusion

In conclusion, given that LGRs of the *MSH2 *gene appear to be frequent in Slovakian HNPCC families, we recommend beginning mutational screening in this population with the MSH2/MLH1-MLPA kit. We have discovered partial LOH events at mutated genes in tumors from the *MLH1/MSH2 *large genomic rearrangement carriers. To the best of our knowledge, this finding is novel. For the fine mapping of conversion tracts in these genes, a large set of heterozygous markers is essential. Our results also extend the limited knowledge of the role of gene conversion in cancer.

## Competing interests

The authors declare that they have no competing interests.

## Authors' contributions

KZ and TS carried out the MLPA analysis. TK and MGB performed DNA sequencing and SNP analyses. KZ and MGB participated in the drafting of the manuscript. DM performed MSI analyses and DI assessed clinico-genetical features of the patients enrolled in the study. ZB conceived of the study, and participated in its design and coordination, as well as in the writing of the manuscript. All authors read and approved the final manuscript version.

## Pre-publication history

The pre-publication history for this paper can be accessed here:

http://www.biomedcentral.com/1471-2407/9/405/prepub

## Supplementary Material

Additional file 1 **LOH analysis by the microsatellite marker D3S1611 in SK-22 patient**. An example of tumor with LOH at the D3S1611 marker, Retention of heterozygosity at the D3S1611 marker in tumor of SK-22 patient. Click here for file

Additional file 2 **Data from LOH analyses by MLPA**. Table of the average dosage quotients of exons affected by LGR in matched normal and tumor DNA of patients with identified LGRs in the *MLH1 *or *MSH2 *genes and calculated LOH ratios. Click here for file

Additional file 3 **Data from LOH analyses by MLPA**. Table of the signal intensities of SNP alleles in matched normal and tumor DNA in the patients with identified LGRs in the *MLH1 *or *MSH2 *genes and calculated LOH ratios. Click here for file
