# Supplementary figures and images for: Partial loss of heterozygosity events at the mutated gene in tumors from MLH1/MSH2 large genomic rearrangement carriers
Source: BMC Cancer. 2009 Nov 20;9:405. doi: 10.1186/1471-2407-9-405 (PMC2788582; doi:10.1186/1471-2407-9-405)

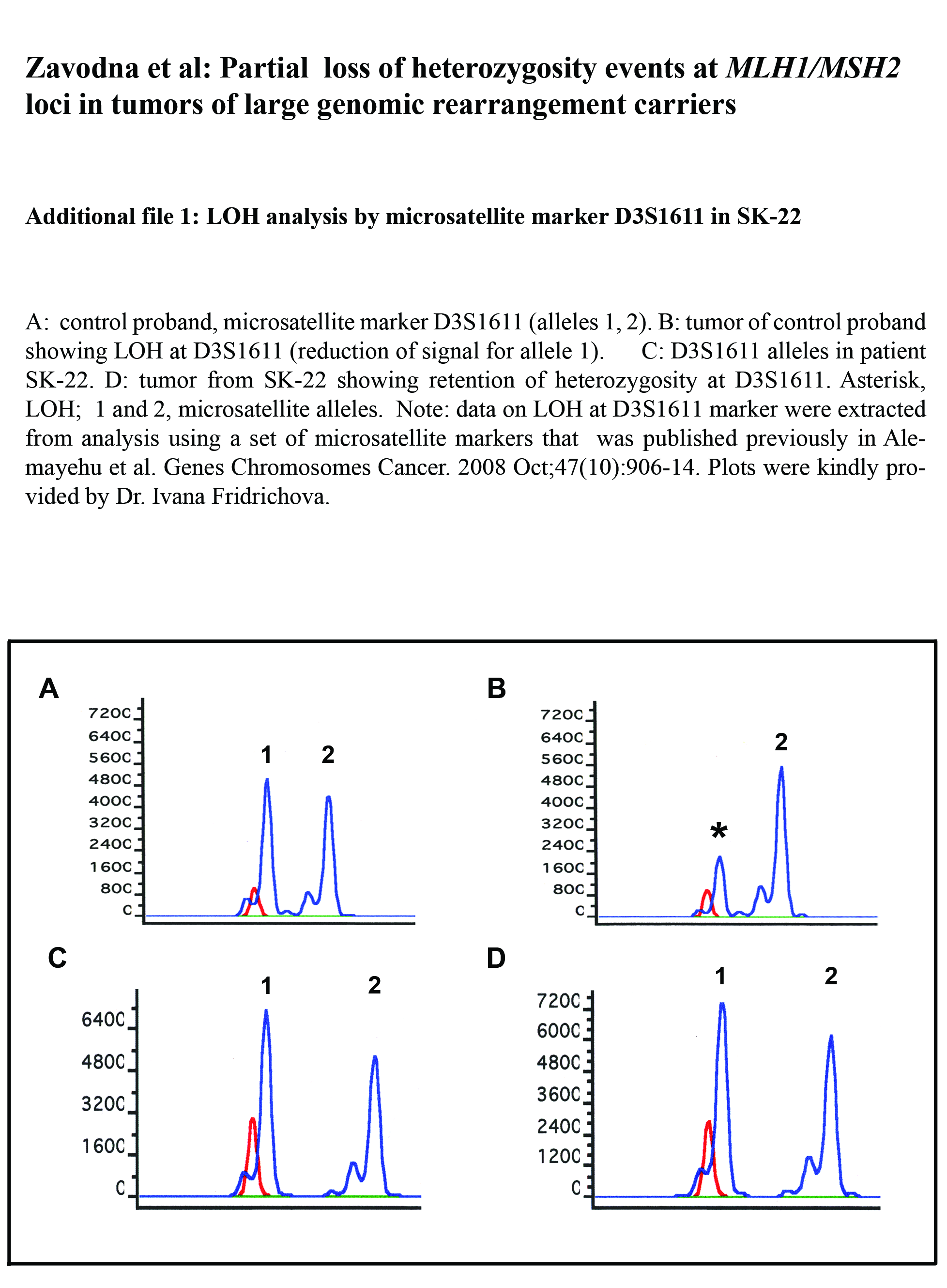

Supplement: Additional file 1 — LOH analysis by the microsatellite marker D3S1611 in SK-22 patient. An example of tumor with LOH at the D3S1611 marker, Retention of heterozygosity at the D3S1611 marker in tumor of SK-22 patient. [file 1471-2407-9-405-S1.TIFF]
