# Supplementary material for: Partial loss of heterozygosity events at the mutated gene in tumors from MLH1/MSH2 large genomic rearrangement carriers
Source: BMC Cancer. 2009 Nov 20;9:405. doi: 10.1186/1471-2407-9-405 (PMC2788582; doi:10.1186/1471-2407-9-405)
Supplement: Additional file 2 — Data from LOH analyses by MLPA. Table of the average dosage quotients of exons affected by LGR in matched normal and tumor DNA of patients with identified LGRs in the MLH1 or MSH2 genes and calculated LOH ratios. [file 1471-2407-9-405-S2.DOC]

**Zavodna et al., Partial loss of heterozygosity events at the mutated gene in tumors from *MLH1/MSH2* large genomic rearrangements carriers**

**Additional file 2: Data from LOH analyses by MLPA**

Ratio implying LOH is in blue. N - average dosage quotient of exons affected by LGR in normal DNA, T - average dosage quotient of exons affected by LGR in tumor DNA.

| **Patient**  **(mutated gene in the germline)** | **N** | **T** | **LOH ratio** |
| --- | --- | --- | --- |
| **SK-22 (*MLH1*)** | 0.55 (±0.039 SD) | 0.20 (±0.02 SD) | 0.36 |
| **SK-14 (*MSH2*)** | 0.54 (±0.03 SD) | 0.49 (±0.13 SD) | 0.90 |
| **SK-20 (*MSH2*)** | 1.57 (±0.08 SD) | 1.67 (±0.02 SD) | 1.06 |
| **SK-21 (*MSH2*)** | 0.67 (±0.12 SD) | 0.77 (±0.14 SD) | 1.15 |
| **SK-23 (*MSH2*)** | 0.65 (±0.07 SD) | 0.56 (±0.17 SD) | 0.86 |
