# Supplementary material for: Partial loss of heterozygosity events at the mutated gene in tumors from MLH1/MSH2 large genomic rearrangement carriers
Source: BMC Cancer. 2009 Nov 20;9:405. doi: 10.1186/1471-2407-9-405 (PMC2788582; doi:10.1186/1471-2407-9-405)
Supplement: Additional file 3 — Data from LOH analyses by MLPA. Table of the signal intensities of SNP alleles in matched normal and tumor DNA in the patients with identified LGRs in the MLH1 or MSH2 genes and calculated LOH ratios. [file 1471-2407-9-405-S3.DOC]

**Zavodna et al., Partial loss of heterozygosity events at the mutated gene in tumors from *MLH1/MSH2* large genomic rearrangements carriers**

**Additional file 3: Data from LOH analyses by the SNP markers**

Ratios implying LOH at respective gene are in blue. N1, N2 - the alleles in normal DNA, T1, T2 - the alleles in tumor DNA.

| **Patient**  **(mutated gene in the germline)** | **SNP marker** | **Gene** | **N1** | **N2** | **T1** | **T2** | **LOH ratio** |
| --- | --- | --- | --- | --- | --- | --- | --- |
| **SK-14 (*MSH2*)** | c.1551-91G>T | *MSH2* | 1286 | 2466 | 1257 | 1130 | 0.468803734 |
|  | c.1551-9A>T | *MSH2* | 366 | 319 | 512 | 184 | 0.412323668 |
|  | c.1661+12G>A | *MSH2* | 341 | 323 | 482 | 204 | 0.44682245 |
|  | c.1759+107A>G | *MSH2* | 450 | 375 | 570 | 230 | 0.484210526 |
|  | c.655A>G | *MLH1* | 3539 | 1933 | 5325 | 2988 | 1.027329336 |
|  | c.1668-19G>A | *MLH1* | 4019 | 3731 | 3776 | 3593 | 1.02498613 |
|  | c.1990-121T>C | *MLH1* | 6212 | 3553 | 3838 | 2292 | 1.044109104 |
| **SK-20 (*MSH2*)** | c.211+9C>G | *MSH2* | 7175 | 2515 | 6592 | 571 | 0.247117115 |
|  | c.211+98C>T | *MSH2* | 6698 | 6625 | 3192 | 1337 | 0.423475008 |
|  | c.1277-118G>A | *MSH2* | 940 | 401 | 1352 | 277 | 0.480271215 |
|  | c.1990-121T>C | *MLH1* | 3572 | 2202 | 3959 | 2189 | 0.896921419 |
|  | c.1039-78G>A | *MLH1* | 2934 | 1807 | 5532 | 3284 | 0.963879922 |
|  | c.1038+8C>T | *MLH1* | 2016 | 1414 | 1880 | 1278 | 0.969201601 |
| **SK-22 (*MLH1*)** | c.1-93G>A | *MLH1* | 356 | 286 | 612 | 161 | 0.327460122 |
|  | c.211+98C>T | *MSH2* | 404 | 382 | 1089 | 1015 | 0.985725893 |
|  | c.2006-6T>C | *MSH2* | 1187 | 508 | 1046 | 521 | 1.163839373 |
|  | c.1661+12G>A | *MSH2* | 935 | 569 | 929 | 528 | 0.933936939 |
| **SK-21 (*MSH2*)** | c.1990-121T>C | *MLH1* | 4230 | 2499 | 6057 | 2615 | 0.730782655 |
|  | c.1039-78G>A | *MLH1* | 5829 | 3558 | 3859 | 2333 | 0.990439773 |
|  | c.1038+86C>T | *MLH1* | 1637 | 1153 | 1999 | 1421 | 1.00925441 |
|  | c.1-93G>A | *MLH1* | 2227 | 1410 | 2294 | 1540 | 1.060299146 |
|  | c.655A>G | *MLH1* | 3290 | 2013 | 2200 | 1398 | 1.038572009 |
|  | c.1551-9T>A | *MSH2* | 1854 | 1312 | 1088 | 997 | 1.294917672 |
|  | c.1661+12G>A | *MSH2* | 2024 | 1087 | 1522 | 975 | 1.192809055 |
| **SK-23 (*MSH2*)** | c.1276+1394A>T | *MSH2* | 997 | 560 | 621 | 408 | 1.169703244 |
